# Katrin Steffen 2021: Blut und Metall. Die transnationalen Wissensräume von Ludwik Hirszfeld und Jan Czochralski im 20. Jahrhundert. Göttingen: Wallstein Verlag, geb., 568 S., 11 Abb., 59,00 €, ISBN: 978-3-8353-5013-7.

**DOI:** 10.1007/s00048-023-00360-3

**Published:** 2023-06-15

**Authors:** Alexej Lochmatow

**Affiliations:** Universität Erfurt, Erfurt, Deutschland

In ihrer Publikation *Blut und Metall* widmet sich Katrin Steffen einer wissenschaftshistorischen Analyse der intellektuellen Wege von zwei „internationalen Wissensakteuren“ (9), nämlich dem Mediziner Ludwik Hirszfeld (1884–1954) und dem Chemiker Jan Czochralski (1885–1953). Hirszfelds Schwerpunkt lag auf der systematischen Blutforschung, durch die er eine neue Disziplin – die Seroanthropologie – begründete. Czochralski beschäftigte sich mit der anorganischen Chemie und wurde zum Entdecker einer ganzen Reihe von Metalllegierungen. Bereits die Auswahl zweier so unterschiedlicher Figuren, die ihre polnische Herkunft und ihre fast identischen Lebenszeiten verbinden, weist auf die methodische Vorgehensweise der Autorin hin. Steffen bezieht sich sowohl auf die klassischen Thesen der Wissenssoziologie à la Ludwik Fleck als auch auf die neuesten Publikationen aus dem Bereich der politischen Epistemologie und betont, dass keine wissenschaftliche Biografie ohne entsprechende Rekonstruktion der relevanten politischen und historischen Kontexte möglich sei. Die Tatsache, dass Hirszfeld und Czochralski einander kaum kannten (10), ist für die Autorin nur ein weiterer Beleg dafür, dass sich eine komparative Perspektive auf ihre wissenschaftlichen Aktivitäten angesichts der Ähnlichkeit ihrer politischen und sozialen Erfahrungen als äußerst produktiv erweist.

Sowohl für den im „preußischen“ Teil Polens geborenen Czochralski als auch für den aus dem „russischen“ Warschau stammenden Hirszfeld war das deutsche Kaiserreich der Ort ihres wissenschaftlichen Werdens und der Erste Weltkrieg ein Labor für die praktische Anwendung ihrer wissenschaftlichen Ideen. Steffen zeigt, dass die beiden Wissenschaftler zu Beginn des Krieges die Grundsteine ihrer späteren wissenschaftlichen Erkenntnisse bereits gelegt hatten. Hirszfeld hatte sich schon vorher im akademischen Bereich etabliert und betrieb international anerkannte Forschungen zur Klassifizierung der Blutgruppen (70–71). Der „Autodidakt“ (63) Czochralski wurde zu einem der führenden Experten des AEG-Konzerns, dessen Forschung zu Metalllegierungen auch unter Fachchemikern anerkannt wurde (83–84). Nach Kriegsbeginn schloß sich Hirszfeld der russischen Armee an. Der deutsche Staatsbürger Czochralski war dagegen in den kriegsorientierten Industriezweigen Deutschlands tätig. Steffen zeigt in diesem Zusammenhang, dass beide Wissenschaftler den Krieg nutzten, um ihre Forschung auf ein neues Niveau zu bringen. Hirszfeld untersuchte den Zusammenhang zwischen Blutgruppen und „Rassen“ anhand des umfangreichen „Materials“ des Bluts von Soldaten und Kriegsgefangenen, was der Entwicklung seiner „seroanthropologischen“ Ideen diente (96–110). Czochralski experimentierte mit verschiedenen militärrelevanten Legierungen, was wiederum den Krieg zur „Geburtsstunde“ des berühmten Czochralski-Verfahrens machte (mit dem Einkristalle aus reinem Silizium gezüchtet werden; 118).

Die Zugehörigkeit von Hirszfeld und Czochralski zum polnischen nationalen Diskurs und ihr Anteil an der imaginären Community der polnischen *Inteligencja* sind ein Leitmotiv des Buches, das erst mit der Beschreibung der Gründung des unabhängigen polnischen Staates in den Vordergrund rückt. Steffen führt an, dass – wenigstens vor der Machtergreifung durch Józef Piłsudski – viele der führenden politischen Figuren des neugegründeten Staates Fachwissenschaftler waren und die Einladung bekannter Spezialisten nach Polen häufig auf der Regierungsebene diskutiert wurde. Diese außerhalb des Kreises von Osteuropaexperten kaum bekannte Tatsache veranschaulicht unter anderem, wie Wissenschaftlerinnen und Wissenschaftler selbst als Treiber der Idee einer nationalen Wissenschaft agierten.

Der Ausbruch des Zweiten Weltkriegs änderte die Konstellation der gesellschaftlichen Faktoren, die die wissenschaftliche Praxis der beiden Wissenschaftler bestimmten, ganz wesentlich. Czochralski konnte seine wissenschaftliche Arbeit offiziell fortsetzten. Hirszfeld musste ins Warschauer Ghetto umsiedeln. Steffen zeigt, dass sowohl ihre polnische Identität als auch ihre internationalen Netzwerke auch unter den Bedingungen der Besetzung durch das nationalsozialistische Deutschland der Forschung dienen konnten. Czochralski brach seine Kontakte nach Deutschland nicht ab und arbeitete an neuen Legierungen für die Wehrmacht, half aber gleichzeitig der polnischen Widerstandsbewegung. Hirszfeld, der sich als Pole verstand, sich jedoch von der jüdischen Identität distanzierte (382), sammelte auch im Ghetto neue Materialien für seine Blutforschung und versuchte das Renommee seiner deutschsprachigen Publikationen zur Rassenkunde dafür zu nutzen, sein Leben zu retten (379).

Die Resistenz der wissenschaftlichen Ideen der beiden Wissenschaftler trotz der politischen Kataklysmen ist für Steffen ein wichtiger Punkt. Die Überzeugung, dass wissenschaftliche Erkenntnisse per se nicht politisch sind (wenngleich Wissenschaft nationalen Zwecken dienen kann), war einer der Faktoren, die die Überlebensfähigkeit ihrer wissenschaftlichen Praxis sicherten. Während Czochralski nach dem Krieg als Nazi-Kollaborateur jedoch seine Position in der polnischen Wissenschaft verlor, konnte Hirszfeld sein „unpolitisches“ Wissen in den Dienst des unter der sowjetischen Patronage gegründeten polnischen Staates stellen. Steffen zeigt, dass Hirszfeld der „rein wissenschaftlichen“ Kategorie der Rasse treu blieb, wenn er damit auch in Konflikt mit dem sowjetischen Lyssenkoismus geriet und Rassismusvorwürfen seitens sowjetischer Kollegen ausgesetzt war.

Erst nach dem Fall des sozialistischen Regimes in Polen wurden die beiden Wissenschaftler für ihre Treue gegenüber dem polnischen Staat und der Wissenschaft gleichermaßen dem nationalen Pantheon zugerechnet, was der kritischen Forschung über ihren wissenschaftlichen Nachlass hohe politische Relevanz verleiht. Steffens Doppelbiografie zeichnet ein enges Netz an Verflechtungen und ist dadurch kaum auf einzelne Thesen reduzierbar. Das Buch macht deutlich, wie jede Biografie als Prisma verschiedenster politischer und sozialer Prozesse seziert werden kann. Basierend auf beeindruckend umfangreichen Archivmaterialien sowie publizierten Quellen versammelt Steffen viele miteinander verflochtene Geschichten, von denen jede einzelne zweifelsohne erzählenswert ist.

